# An Area‐Specific, International Community‐Led Approach to Understanding and Addressing Equality, Diversity, and Inclusion Issues within Supramolecular Chemistry

**DOI:** 10.1002/anie.202015297

**Published:** 2021-03-08

**Authors:** Claudia Caltagirone, Emily R. Draper, Michaele J. Hardie, Cally J. E. Haynes, Jennifer R. Hiscock, Katrina A. Jolliffe, Marion Kieffer, Anna J. McConnell, Jennifer S. Leigh

**Affiliations:** ^1^ Centre for the study of higher education and the School of Physical Sciences University of Kent Canterbury UK; ^2^ Department of Chemical and Geological Science University of Cagliari S.S. 554 Bivio per Sestu 09042 Monserrato (CA) Italy; ^3^ School of Chemistry University of Glasgow Glasgow UK; ^4^ School of Chemistry University of Leeds Leeds LS2 9JT UK; ^5^ Department of Chemistry University College London 20 Gordon Street London WC1H 0AJ UK; ^6^ School of Chemistry The University of Sydney Sydney 2006 NSW Australia; ^7^ School of Chemistry University of Bristol Cantock's Close Bristol BS8 1TS UK; ^8^ Otto Diels Institute of Organic Chemistry Christian-Albrechts-Universität zu Kiel Kiel Germany

**Keywords:** DEI, EDI, gender, marginalisation, supramolecular chemistry

## Abstract

Diversity, equality, and inclusion (DEI/EDI) are pressing issues in chemistry and the natural sciences. In this Essay we share how an area‐specific approach is “calling in” the community so that it can act to address EDI issues, and support those who are marginalised. Women In Supramolecular Chemistry (WISC) is an international network that aims to support equality, diversity, and inclusion within supramolecular chemistry. WISC has taken a field‐specific approach using qualitative research methods with scientists to identify the support that is needed and the problems the supramolecular community needs to address. Herein, we present survey data from the community which highlight the barriers that are faced by those who take career breaks for any reason, a common example is maternity leave, and the importance of mentoring to aid progression post‐PhD. In conclusion, we set out an interdisciplinary and creative approach to addressing EDI issues within supramolecular chemistry.

## Graphical abstract

Marginalisation can be experienced for many intersectional reasons including race, ethnicity, sexuality, religion, and gender. This Essay sets out a field‐specific approach to supporting equality, diversity, and inclusion in supramolecular chemistry as pioneered by an international network of Women In Supramolecular Chemistry (WISC). WISC aims to “call in” the community and to use qualitative approaches to set out a framework for addressing EDI.

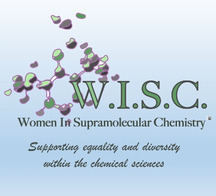

Leading figures in the scientific and chemistry community are asking for conversations on Diversity, Equality,^1^ and Inclusion (DEI/EDI), and the accompanying actions that will achieve change to be brought into the mainstream.[^1a^, ^1b^] A slew of chemistry editorials have appeared setting out the need for the discipline to address issues of sexism and racism, and to move beyond words and into action.[^2a^, ^2b^, ^2c^, ^2d^] In this Essay we will share how, in one field—Supramolecular Chemistry—we have initiated a new network that is listening to the needs of the community, then bringing this community together to support marginalised scientists. Marginalisation can come about for a multitude of reasons, and within academia it is often thought to correlate with characteristics of the individual such as colour, ethnicity, disability, class, and access.[^3a^, ^3b^] In terms of gender, it is well‐established that women in academia are disproportionately affected by funding structures, academic culture, research environments, and caring responsibilities, and indeed a body of work exists on academic identity and women's lived experiences as they negotiate and resist structural inequalities.[^4a^, ^4b^, ^4c^] The COVID‐19 pandemic and lockdown has exacerbated these gender differences.[^5a^, ^5b^]n1

Within chemistry, the gender gap is among the widest of the STEM disciplines. For example, between 2011 and 2019 there was only a 1 % increase in the proportion of female invited speakers to a group of chemistry conferences.[^6a^, ^6b^] Over the same time period, the proportion of female speakers increased from 7 % to 38 % in Artificial Intelligence and Machine Learning.[^6a^, ^6b^] The lack of retention and progression for women and all those with protected EDI characteristics is pronounced.[^7a^, ^7b^] For example, the proportion of women choosing to study chemistry as undergraduates in the UK was 45 % in 2014/2015 compared to 20 % choosing physics, and yet, in both, the proportion of women at professorial levels was just 9 %.^[7b]^ Given these statistics, this may lead to some women who are full professors gaining more exposure, due to invited speaker presentations, than their male counterparts. However, more women are employed on fixed‐term precarious contracts.^[7b]^ Women author fewer papers, are cited less, and face bias at every stage of the publication process.^[8]^ Proportionately fewer women sit on editorial boards, are nominated for awards, and far fewer file patent applications.[^7a^, ^7b^, ^8^] Sexual harassment abounds on campuses,^[9]^ with 58 % of women in academia having faced some kind of sexual harassment:^[10]^ “*most female scientists have personal stories of things that have happened to them*”.^[11]^ Gender is of course not the only factor that those in STEM face. When this is looked at intersectionally and we consider the impact of culture and other factors such as disability, ethnicity, and race, these barriers compound, contributing to a much larger overall effect.^[12]^ Women of colour are more likely to experience higher levels of harassment than white women.^[10]^ These harassments contribute to a culture in chemistry where those from minority groups are more likely to leave the field, leading to a lack of diversity “*science in this era [is] essentially dominated by white men… even to this day Frances Arnold is only the 5th woman to receive the Chemistry Nobel Prize, and there have been no black chemistry laureates”*.^[2a]^ There is some evidence of recent change: in October 2020, Emmanuelle Charpentier and Jennifer Doudna became the 6th and 7th women to be awarded the Nobel Prize in Chemistry for their development of a method for genome editing.^[13]^ Three out of the last 10 chemistry laureates have been women. However, since 1901, there have been 112 Nobel Prizes awarded in Chemistry, to 185 individuals,^[14]^ so less than 4 % have been awarded to women, and none to a black scholar. In 2019 the Royal Society of Chemistry said that at the current rate of change, the chemical sciences in the UK will never reach gender parity.^[7b]^ Therefore, in chemistry there is no argument—women are a marginalised group.

Women in Supramolecular Chemistry (WISC) is an area‐specific international community initiated in 2019 by the authors to support equality and diversity in the chemical sciences. As such, it sets out to “call‐in”^[15]^ members of the community, so that the community can act to address EDI issues, and support those who are marginalised by gender and other characteristics. Whereas “calling out” usually refers to publicly pointing out oppressive behaviour, “calling in” is the gentler act of alerting peers to their behaviour with compassion and guidance. Both acts aim to stop oppression. WISC is led by an international team of women from early to mid‐career levels, with an advisory board comprising diverse and senior researchers in the field. As a cohort, we represent researchers from four continents, different ethnic backgrounds, and include members with disabilities/chronic illnesses/neurodivergencies. At a general early career event in the community, the leap from post‐doctoral researcher (post‐doc/PDRA) to independent researcher was identified as a career transition in need of support. The post‐PhD period (typically when a researcher is in their 20s/30s) has been identified as a time when large numbers of women leave science.^[7b]^ Progression and promotion opportunities for post‐docs are limited in all disciplines.^[16]^ Although having a post‐doctoral position is often seen as a required stage in the development of a successful academic career,^[17]^ they do not always have a clear, adequately supported pathway to future career progression.^[18]^ The typically short‐term contracts that post‐docs have, coupled with the requirement to undertake more than one position and changing institution/location can mean that researchers in their late 20s/early 30s are expected to uproot their personal lives for work commitments. This is difficult for everyone; however it is particularly challenging for those who need a stable network for childcare, other caring or family commitments, or mental health.^[19]^ It can easily be forgotten that a supportive atmosphere is often the most important criterion in enhancing life satisfaction and maintaining positivity about a research career for post‐docs.^[16]^ It has been recognised that research culture in chemistry^[20]^ and across wider research is far from ideal.^[21]^ Resilience is often talked about within academia;[^22a^, ^22b^, ^22c^] however there is little written about how we can culture resilience through appropriate disciplinary community support and culture changes.

This Essay presents findings from a survey carried out, with the aim of sharing lived experiences that help to identify areas that the community felt were problematic and that WISC could support. Moving forward, WISC has chosen to adopt a methodology which prioritises a qualitative approach to aspects of embodied lived experiences such as sharing stories, reflecting, and processing events, and using these as a basis of research. Qualitative research uses non‐numerical data to understand concepts, opinions, and experiences and to gain in‐depth insight into an issue.^[23]^ Although quantitative methods more traditionally used within science can tell us whether there is a problem and the extent to which it affects people; qualitative research can be used to “*change the world… [Qualitative researchers] are challenged to confront the facts of injustice, to make the injustices of history visible, and hence open to change and transformation”*.^[24]^ Put simply, quantitative research can tell us the scale of a problem and that there is a need to change. Qualitative research and social science methods are able to humanise the reasons why this work is important and help to design the approaches to effect this change. Marginalisation is a feeling not easily captured by numbers. Collaborating with experts in these research methods ensures that the work is carried out with rigour and validity. If we need to ensure there is action in addition to words as we combat EDI issues in chemistry, then it makes sense to utilise the research experts who use tools designed to do this.

## Demographic Data

**Figure 1:**
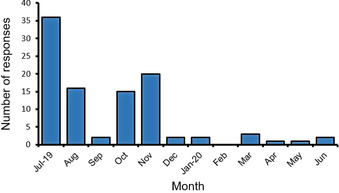
Histogram showing the numbers of survey responses by month from July 2019–June 2020.

As the *“community working to support the community”* is a central WISC ethos, surveying the supramolecular community to direct the network's approach to *“supporting equality and diversity within science”* was vital. The survey was opened for responses in July 2019, and Figure 1 shows the numbers of responses by month, reaching 100 in June 2020, with spikes in response numbers correlating with conferences/events. Of these 100 responses, 81 % were female, and the distribution of career stages was as shown in Table 1.

**Table 1:** Career stages of respondents [%].

Masters Student	PhD Student	PDRA	Research Fellow	Independent Researcher	Other^[a]^
5	30	24	2	39	4

[a] Other was explained as lecturer, professor, enrolled for PhD, pharmacist.

The range of ethnicities self‐declared by these respondents in an open comment box included: Asian, Japanese, Chinese, Pakistani, African, Mixed, Persian, French/Moroccan, Black, Indian, Middle Eastern, German, European, and Hispanic. However, the majority identified as white and European. The country of origin, and the country the respondent was based in, was not recorded.

Of these respondents, 10 % self‐identified in an open comment box as having an EDI‐protected characteristic, and these included disability, LGTBQIA+, age, and religion. In addition, some respondents stated that being a mother, working part‐time, and being an immigrant were EDI characteristics.

## Transition to Independent Researcher and Mentoring

WISC′s survey data show that within the community of supramolecular chemists there is a desire for support to make the transition from post‐doc to independent researcher, that mentoring is seen as valuable and desirable, and that there is a need for a space to talk about and share concerns around career breaks, parenting, and the demands of balancing work with other aspects of life.

These women respondents spoke about the difficulties transitioning to independence in chemistry:

- “*I would like to see the progression of women scientists establishing independent Labs in the field of supramolecular chemistry*.”
- “*Transition from pdra [postdoc] to lecturer is a key challenge. Focus on supporting/encouraging women in applying for positions*.”
- “*Having resources and mentors are exceptionally important. I find myself seeking advice from those who have been in my shoes. I think PhD and postdoc students could use this mentoring from early career independent researchers, and early career researchers would benefit from those more experienced*.”

Women respondents continued to speak about the importance of mentoring for progression and retention of women:

- “*From personal experience I have found that regular mentoring and support makes a world of difference in terms of career development. As a female in science, there are often other factors to consider, such as family constraints and having support from other women who are in a similar circumstance may be advantageous*.”
- “*Advice, knowing their route into supramolecular chemistry, mentoring sounds really useful too :‐)*”

As a result of these data, WISC set up an area‐specific mentoring scheme, where small groups of peers support each other and are linked to a senior researcher. This scheme has learnt from other initiatives such as the Chemistry Women Mentorship Network.^[25]^ Mentors are at least one career‐stage further ahead of a small group of mentees (e.g. post‐docs mentor PhD students, early career independent researchers mentor post‐docs etc.). Mentors are not necessarily women, as WISC looks to the community to support others, although there is a balance of different institutions and geographical spread in each cluster. Further details on WISC′s mentoring opportunities can be found on the WISC website.^[28]^ The area‐specific nature of the WISC scheme means that mentees interact with researchers senior to them, who are able to advise directly on the opportunities and challenges particular to supramolecular chemistry.

## Career Breaks

In answer to a specific question, 30 % said they had taken a career break. Respondents who had not taken a career break were worried about the potential impact. These women said:

- “*I do not anticipate a break (but things do happen!). I would worry about re‐entering academia after a break, and finding it difficult to catch back up/missing out on things (conferences, workshops, funding cycles, etc)*.”
- “*I am quite scared about having kids before getting permanent/more stable*.”
- “*From past hearsay and departments I′ve worked in, it's almost as though women who have taken career breaks in chemistry seem to just fall off the radar, with no support from the department, and that those that really push forwards with their career are seen to be really ”pushy“ or ”over‐reaching“ which is awful! Needs to change*.”

Women respondents who had taken a career break shared their experiences:

- “*Support is patchy, expectations are wildly different, and I lost a first authorship which may have affected how people perceive my career. Some in the community are incredibly supportive. Some less so*.”
- “*I started as a professor immediately after finishing mat [sic] leave, so I didn't have work to catch up. Overall, my transition was smooth. However, reviewers DON′T SEEM TO PAY ATTENTION to the ”Leave of Absence“ entry in the CV. on my grant review, only 1/3 reviewers acknowledge the leave*.”
- “*On returning I found I was behind on my research and unsupported*.”

Although comments were mixed overall, parental leave is unequal.^[26]^ In contrast to the women, respondents identifying as white men largely had positive experiences:

- “*2×3 months for each of my children. No problems returning as yet, but I did get comments in grants about ”gaps“ in publication records*!”
- “*Half a year parental leave. No troubles returning, at least that I noticed*.”
- “*My return was smooth, as this was managed within my contract as a faculty member*.”
- “*Once I returned, all of my former colleagues and peers were amazingly supportive*.”

Many women reported working whilst on leave, followed by challenges on return:

- “*Maternity leave, about 6 months, all spent writing papers/reviews not to lose the pace, so really not quite a break. I was advised by my mentor that if I really did have a break, I would be left behind*.”
- “*2×1 year break, I was able to return without too much trouble. Luckily I won grants while on maternity leave. It was a bit tricky to get lab space and office space back. I haven't been to many conferences in the last 6 years. This is challenging when pregnant/with small kids*.”
- “*16 weeks, maternity leave. The return was easy in my case, since I dealt with projects during my break*.”

The expectation of having to work whilst on leave, combined with the physical processes and sometimes associated complications of birth and breastfeeding; along with ingrained gender stereotypes that leave women shouldering much of the work around childcare and domestic chores may help to explain the differences in experiences between men and women taking career breaks or parental leave. The impact of COVID‐19 has exacerbated these gender differences.[^5a^, ^5b^, ^27a^, ^27b^] WISC′s second survey^[28]^ (launched October 2020) is seeking to collect data on these experiences for those working in laboratories and in supramolecular chemistry.

## Willingness to Support and Ideas for the Future

When asked about future activities, there was interest in everything that WISC suggested, with at least 51–62 % indicating interest in reading blog posts; attending network meetings; being matched with a mentor; attending a forum and using reflective/creative approaches to explore experiences; acting as a mentor to someone at an earlier career stage; receiving updates on funding opportunities; attending skills‐based workshops; and attending panel discussions with early to senior career researchers with questions from the audience.

Many respondents offered additional comments in support of WISC, and its aims:

- “*This is a wonderful initiative and I would be absolutely delighted to contribute to it at any capacity*!”
- “*There is a shortage of role models for women. I have participated in conferences with 1 % of women as speakers, it is clear that women are underrepresented. We need to support each other through experience sharing and make each other visible, at least among ourselves, to be able to promote each other*.”
- “*Continue with the great ideas! It would be good to discuss issues surrounding the effects of parental leave on careers and how to minimise this, particularly with respect to getting funding bodies on board with these adaptations*.”

The field‐specific nature of WISC has meant that the activities bourne out of the survey results have been supported at conferences for the community through panel sessions at these events and the like. In response to the survey results, WISC has set up a field‐specific mentoring scheme and a Parenting Cluster, which is a protected space for men, women, and non‐binary folx^[29]^ to talk about a variety of topics, including the pressures of parenting, step‐parenting, and fertility issues.

Women in STEM are subject to different pressures than those in other disciplines of academia. Although there is a large body of work dedicated to the development of marginalised identity in academia,[^3a^, ^4a^, ^4c^, ^30^] the work dedicated to tackling diversity and underrepresentation in STEM is far smaller.[^31a^, ^31b^, ^31c^] There are many reasons for this, not least that the nature of learning and knowledge and the types of skills developed in STEM rarely includes reflective practices. This in turn impacts on how women and marginalised groups construct their identity, and process and disseminate their experiences. In contrast, when researching into lived experiences, it is common to use reflective and qualitative research methods and approaches such as interviews, ethnography and auto‐ethnography.[^3a^, ^32a^, ^32b^] Even within disciplines that are accustomed to writing reflectively, “*there were very few women academics who talked openly about their personal lives […] I began to see academia as the (stereotypically) masculine and isolated place it was designed to be—a place free from children, romantic relationships, personal problems and lives*”.^[33]^ If the stories and experiences of those who do not fit the stereotype are not visible, then it is harder for those outside the majority group to feel they belong. Individuals do not always share their stories of marginalisation publicly for a multitude of reasons, including the fear that it may adversely affect their career. Yet it is important for all within STEM to “*understand that nearly every one of your colleagues who is a member of an underrepresented group (women, BIPOC) has been told at some point in their career that they only received a position, fellowship, award, or invitation because of their minority status*”.^[2d]^

Moving forward, WISC has adopted a transdisciplinary^[34]^ ethos. WISC has the unique opportunity to trailblaze this approach, as their social scientist board member is herself a statistic of the failure of chemistry to support the retention of women. She wrote “*My first degree is in Chemistry with Analytical Science. I completed 2* 1/2 *years of a PhD in Computational Chemistry before life took me in a different direction”*.^[35]^ She is positioned as an insider^[36]^ rather than a social scientist performing research on scientists. Her social science research on areas such as marginalised academics, reflective practice, and embodied academic identity[^37a^, ^37b^, ^37c^, ^37d^] feed into the aims and intentions of the network. Additionally, she is an expert in creative approaches to qualitative research,^[38]^ which are particularly suited where the issues of exploration are sensitive, as in the case here, as there are human stories behind every statistic of non‐retention. Scientists are not always knowledgeable of, or willing to participate in, social science or artistic research.^[39]^ With the addition of a trusted‐insider social scientist using arts‐based methods it becomes possible to do more and use qualitative research approaches to cast light on and process hitherto‐unseen lived experiences. Current projects include the use of rhythmanalysis,^[40]^ creative workshops,^[41]^ auto‐ethnography,^[32a]^ and visual ethnography^[42]^ in addition to more conventional surveys and interviews. We believe this approach of engaging chemists with innovative research techniques more commonly associated with Social Sciences and Arts is unique. By collaborating with an expert in social science, our work on marginalisation and EDI ensures we have appropriate ethical approval and gather the data needed to answer specific research questions. In addition, when the answers we receive border on emotional experiences of infertility, step‐parenting, imposter syndrome, and post‐natal depression, the expertise in dealing with qualitative research into sensitive subjects such as identity development means that we are prepared and able to support both researchers and participants.

## Summary

WISC has taken a novel approach to addressing EDI issues within supramolecular chemistry. It is distinctive for two reasons. Firstly, it is a field‐specific initiative, with actions that have been designed to fit the stated needs of and are supported by the supramolecular community. Secondly, it involves those with experience in social science and EDI research at every stage, and is intentionally interdisciplinary in its methods and approach. To tackle the lack of diversity in chemistry, it is important to draw on the existing work around EDI in academia as a whole. However, this work cannot be transplanted wholescale into chemistry, as it is not always representative of the specific culture found within particular fields. In addition to the general issues faced by all academics with regard to structural inequalities, overwork, and work‐life balance,[^43a^, ^43b^, ^43c^, ^43d^] it is important to acknowledge the specific demands and context in chemistry. To fit in those who have been marginalised or excluded, we need to change the structures that have been excluding them.^[44]^ In supporting EDI in supramolecular chemistry, WISC was cognisant of including within its committees individuals who have expertise researching into and including those who are marginalised in academia. As such, this area‐specific approach demonstrates that the idea of “calling in” the community to support its own can work. The ongoing progress on the various WISC activities can be found on the WISC website.^[28]^ Combining social science with scientists ensures that the work that is carried out humanises the reasons why this work is important, highlighting the parity and diversity of experience both men and women face whilst maintaining the rigour and validity of the research within the scientific community and beyond. This Essay highlights and shares the lived experiences of men and women from within the supramolecular community. WISC′s aim is for the supramolecular community to support those marginalised within it, and to increase the retention and progression of these individuals post‐PhD. WISC′s hope is that our approach and work may act as a framework to tackle EDI within other fields of chemistry, and wider STEM disciplines.

## Methods

Permissions were obtained from the relevant local authorities: ethical approval from the Centre for the Study of Higher Education, University of Kent, UK, ethical approval number 24062019. WISC opened a 10‐question survey in July 2019. Informed consent was given by all participants of the survey. The survey was open to all and advertised on Twitter and at supramolecular chemistry conferences. The survey was hosted on a free version of SurveyMonkey, which only allowed 100 responses. The survey was closed in June 2020.

All responses were anonymised to protect the identity of the respondents. The data do not show where the respondents were based. The data were seen only by the research team. The data were thematically analysed,^[45]^ and quotations are used to illustrate the main themes as identified by the researchers.

## Conflict of interest

The authors declare no conflict of interest.

## Biographical Information

*Claudia Caltagirone obtained her PhD in Chemistry in 2006 under the supervision of Prof. Vito Lippoli at the University of Cagliari (Italy). After two years as a visiting scientist in the group of Prof. Philip A. Gale at the University of Southampton (UK) she returned to Cagliari, where she is currently Associate Professor. Her research interest mainly focuses on the development of supramolecular architectures based on weak interactions for sensing applications*.

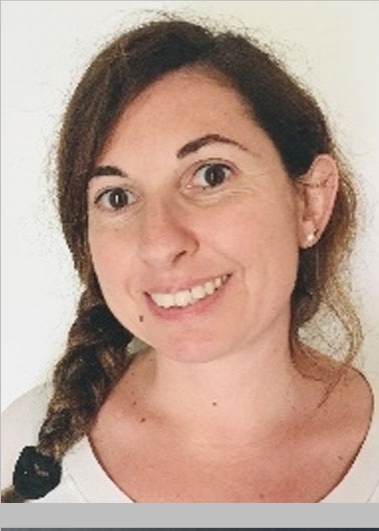

## Biographical Information

*Emily Draper received her PhD from the University of Liverpool in 2015 with Prof. Dave Adams. She carried out post‐doctoral research at Liverpool and then at the University of Glasgow. She became a Leverhulme Trust Early Career Fellow and a Lord Kelvin Adam Smith Fellow in 2017. She took up a Lectureship position at Glasgow in 2018, working on self‐assembled organic electronics before taking maternity leave in 2019*.

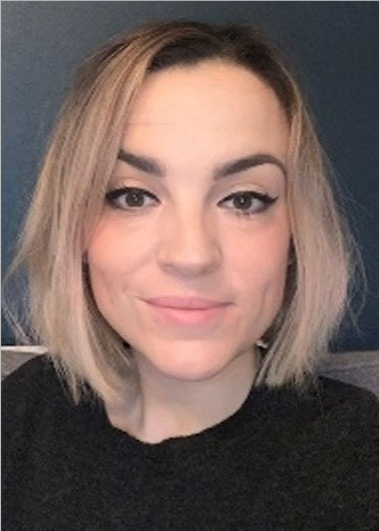

## Biographical Information

*Michaele Hardie completed a PhD in chemistry under the supervision of Profs. Richard Robson and Bernard Hoskins at the University of Melbourne (1996). After several post‐doctoral appointments including at the University of Toledo (USA) and Monash University (Australia), she was appointed lecturer in inorganic chemistry at the University of Leeds (2001), where she is now Professor of Supramolecular Chemistry. Her research interests include coordination and metallo‐supramolecular chemistry of host‐type ligands, coordination polymers, and crystal engineering*.

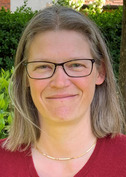

## Biographical Information

*Cally Haynes received a PhD from the University of Southampton in 2013 with Prof. Philip A. Gale. Following post‐doctoral work in Southampton she was appointed as a Publishing Editor at the Royal Society of Chemistry in 2013, and returned to academia in 2015 as a post‐doctoral researcher at the University of Cambridge. She was appointed as a Lecturer in Organic Chemistry and Chemical Biology at UCL in 2019. Her research interests are in molecular transport and the study of self‐assembly processes*.

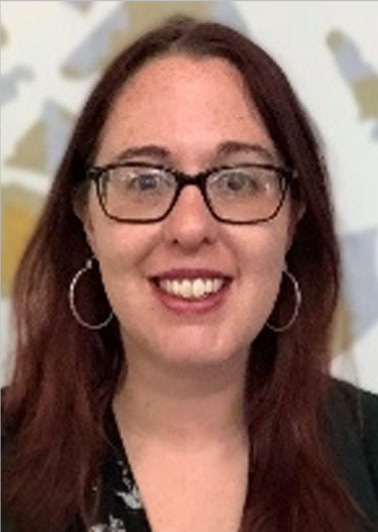

## Biographical Information

*Jennifer Hiscock obtained her PhD in Chemistry (2010) from the University of Southampton (UK) with Prof. Philip A. Gale. Following post‐doctoral work, she moved to the University of Kent (UK) in 2015 as the Caldin Research Fellow, this was followed by her appointment as Lecturer in Chemistry (2016) and Reader in Supramolecular Chemistry (2019). She is now a UKRI Future Leaders Fellow and her current research interests relate to the development of antimicrobial compounds*.

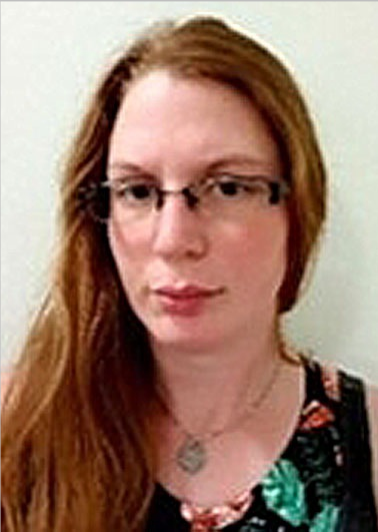

## Biographical Information

*Katrina (Kate) Jolliffe received her BSc (1993) and PhD (1997) from the University of New South Wales with Prof. Michael Padden‐Row. She held positions at Twente University (The Netherlands), the University of Nottingham (UK), and the Australian National University, before moving to the University of Sydney in 2002, where her current position is Payne‐Scott Professor. She is a Fellow of the Australian Academy of Science. Her research interests focus on the design and synthesis of functional molecules*.

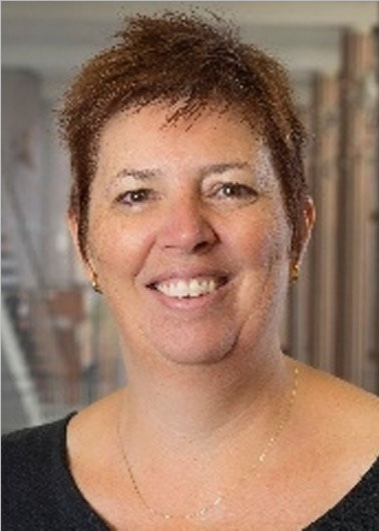

## Biographical Information

*Marion Kieffer obtained her diplome d'ingenieur from the ESPCI ParisTech in 2014. She then completed an MPhil (2015) and PhD (2019) at the University of Cambridge under the supervison of Prof. Jonathan Nitschke. After carrying out post‐doctoral work at the University of Bristol in the group of Prof. Anthony Davis, she joined InnoMedica as an R&D scientist in 2020. Her research interests are in the application of supramolecular chemistry for drug delivery*.

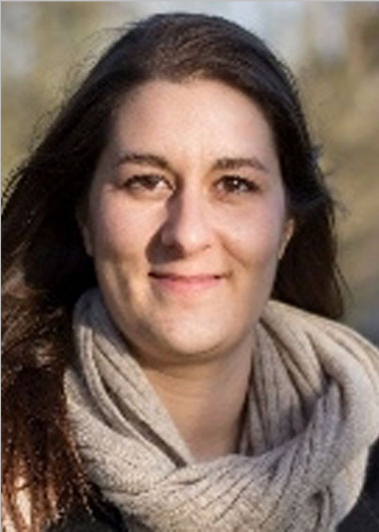

## Biographical Information

*Anna McConnell obtained a D.Phil. under the supervision of Prof. Paul Beer at the University of Oxford. Following post‐doctoral research stays at the California Institute of Technology and the University of Cambridge in the groups of Prof. Jacqueline Barton and Prof. Jonathan Nitschke, respectively, she became a Junior Professor at Christian‐Albrechts‐Universität zu Kiel in November 2016. Her research focuses on stimuli‐responsive metal‐organic cages, dynamic covalent chemistry, and luminescent complexes*.

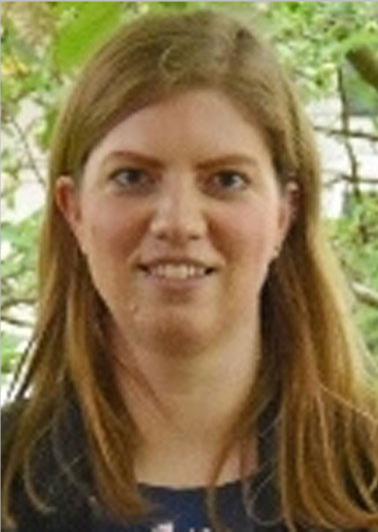

## Biographical Information

*Jennifer Leigh initially trained as a chemist before completing her doctorate in education at the University of Birmingham (2012). She is currently a Senior Lecturer in Higher Education and Academic Practice at the University of Kent (UK). Her research interests include marginalisation in academia, academic practice, academic development, and ableism as well as phenomenological and creative research methods in higher education*.

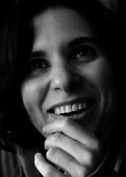

## Footnotes

n1 The E here has been denoted by “equality”, as that is the term used by the UK government and many UK Higher Education Institutions. However, it should be noted that it can also refer to “equity”. Equity is providing various levels of support or assistance, depending on specific needs or abilities.
